# Hyaluronic Acid Levels Predict Risk of Hepatic Encephalopathy and Liver-Related Death in HIV/Viral Hepatitis Coinfected Patients

**DOI:** 10.1371/journal.pone.0064283

**Published:** 2013-05-27

**Authors:** Lars Peters, Amanda Mocroft, Vincent Soriano, Jürgen Rockstroh, Andri Rauch, Anders Karlsson, Brygida Knysz, Christian Pradier, Kai Zilmer, Jens D. Lundgren, for EuroSIDA in EuroCoord

**Affiliations:** 1 Copenhagen HIV Programme, University of Copenhagen, Copenhagen, Denmark; 2 Department of Infectious Diseases, Rigshospitalet, Copenhagen, Denmark; 3 University College Medical School, Royal Free Campus, London, United Kingdom; 4 Hospital Carlos III, Madrid, Spain; 5 University of Bonn, Bonn, Germany; 6 University Clinic of Infectious Diseases, University Hospital Bern and University of Bern, Bern, Switzerland; 7 Venhälsan, Södersjukhuset, Stockholm, Sweden; 8 Department of Infectious Diseases, Wroclaw Medical University, Wroclaw, Poland; 9 CHU Nice Hopital de ĺArchet 1, Nice, France; 10 West-Tallin Central Hospital, Tallinn, Estonia; University of Cincinnati College of Medicine, United States of America

## Abstract

**Background:**

Whereas it is well established that various soluble biomarkers can predict level of liver fibrosis, their ability to predict liver-related clinical outcomes is less clearly established, in particular among HIV/viral hepatitis co-infected persons. We investigated plasma hyaluronic acid’s (HA) ability to predict risk of liver-related events (LRE; hepatic coma or liver-related death) in the EuroSIDA study.

**Methods:**

Patients included were positive for anti-HCV and/or HBsAg with at least one available plasma sample. The earliest collected plasma sample was tested for HA (normal range 0–75 ng/mL) and levels were associated with risk of LRE. Change in HA per year of follow-up was estimated after measuring HA levels in latest sample before the LRE for those experiencing this outcome (cases) and in a random selection of one sixth of the remaining patients (controls).

**Results:**

During a median of 8.2 years of follow-up, 84/1252 (6.7%) patients developed a LRE. Baseline median (IQR) HA in those without and with a LRE was 31.8 (17.2–62.6) and 221.6 ng/mL (74.9–611.3), respectively (p<0.0001). After adjustment, HA levels predicted risk of contracting a LRE; incidence rate ratios for HA levels 75–250 or ≥250 vs. <75 ng/mL were 5.22 (95% CI 2.86–9.26, p<0.0007) and 28.22 (95% CI 14.95–46.00, p<0.0001), respectively. Median HA levels increased substantially prior to developing a LRE (107.6 ng/mL, IQR 0.8 to 251.1), but remained stable for controls (1.0 ng/mL, IQR –5.1 to 8.2), (p<0.0001 comparing cases and controls), and greater increases predicted risk of a LRE in adjusted models (p<0.001).

**Conclusions:**

An elevated level of plasma HA, particularly if the level further increases over time, substantially increases the risk of contracting LRE over the next five years. HA is an inexpensive, standardized and non-invasive supplement to other methods aimed at identifying HIV/viral hepatitis co-infected patients at risk of hepatic complications.

## Introduction

The natural history of liver fibrosis progression in patients with chronic hepatitis B (HBV) or C virus (HCV) infection is highly variable and depends on both host and viral factors [1]. Individuals co-infected with HIV have accelerated progression of fibrosis compared to those with hepatitis mono-infection only [2], [3], and hence requires closer monitoring.

Historically, liver biopsy has been considered the gold standard to diagnose and monitor the progression of fibrosis in patients with chronic viral hepatitis and other liver diseases. However, liver biopsy is an invasive procedure, associated with a small risk of complications and death [4], considerable sampling variability as well as inter- and intra observer variability in the histological staging of the biopsy [5]. Outside clinical trials liver biopsy is not performed in a large proportion of co-infected patients and repeated liver biopsies would be unacceptable for most patients [6], [7].

In recent years a high number of serum biomarkers and other non-invasive methods such as fibroscanning, as markers of liver fibrosis, have been evaluated. Most of these studies have assessed associations of these markers with findings from liver biopsy taken at the same time [8]. Although insightful, this line of investigation suffers from two major limitations. Firstly, the cross-sectional design does not allow for studies of temporal changes in biomarker levels. Secondly, these studies do not inform the evaluation of whether the markers are able to predict relevant clinical end points like hepatic decompensation or death. Mehta et al reported that due to the intrinsic limitations of liver biopsy, the use of this method as a comparator makes it impossible to distinguish a perfectly predictive non-invasive marker from a marker with unacceptable poor predictive potential [9]. Long-term prospective studies of non-invasive methods evaluated against relevant clinical end points are hence required to further advance this research field.

One biomarker, hyaluronic acid (HA) is a component of the extra cellular matrix and primarily cleared from the bloodstream by the hepatic sinusoids [10]. Liver disease will therefore reduce the rate of clearance of HA resulting in elevated plasma levels. This biomarker is attractive to evaluate as it is easy, reliable, inexpensive and freely available to measure. Prior studies, primarily in HCV mono-infected patients, have reported that HA is an accurate individual marker of fibrosis and predictor of hepatic complications [11]–[18]. In HIV, viral hepatitis is a frequent co-infection, and several HIV-specific factors may affect hepatic metabolism. Therefore, the aim of this study was to further evaluate the predictive potential of HA in a large and well characterized cohort of HIV/viral hepatitis co-infected patients followed over extended periods of time within the EuroSIDA study.

## Methods

### Ethics Statement

Before any study related activities are performed Local Ethical Committee approval of the study and procedure for obtaining informed consent from participants is obtained according to local and/or national regulations in all countries participating in the study as well as other national regulatory approvals as applicable. The senior investigator at each clinical site is responsible for obtaining and maintaining this/these approval(s) at all times during the conduct of the study.

#### Patients

The EuroSIDA study is a prospective, observational cohort of 18,277 HIV-1–infected patients in 108 centers across Europe, Israel, and Argentina. The study has been described in detail previously [19]. In brief, patients were enrolled into 9 cohorts from May 1994 onward and median follow-up time is to December 2011. At recruitment, in addition to demographic and clinical information, a complete antiretroviral treatment history is obtained, together with the most recent CD4 cell count and plasma HIV-RNA measurements. At each follow-up visit, details on all CD4 cell counts and plasma HIV-RNA values measured since the last follow-up visit are extracted, as are the dates of starting and stopping each antiretroviral drug received and the use of drugs for prophylaxis against opportunistic infections. The dates of diagnosis of all AIDS-defining illnesses, non–AIDS-defining malignancies, and other serious infections are also recorded. Anti-HCV antibody and hepatitis B surface antigen (HBsAg) status was collected in 1997 for the recruitment of the third cohort, and for all patients from the first two cohorts who remained under follow-up at the date. Since 1997 hepatitis serology and virology have been updated annually.

The EuroSIDA plasma repository was set up in 1997 and collects plasma samples from all patients at 6 months intervals when patients are seen for their regular outpatient visits. Samples are stored at –80°Celsius. Patients with unknown HBsAg or HCV serostatus and with stored plasma samples were identified in 2006 and anti-HCV IgG and HBsAg in these samples were determined. Plasma HCV-RNA was quantified in all anti-HCV antibody positive samples using the Versant HCV-RNA v3.0 assay (Bayer Diagnostics, Berkeley, CA), which has a lower limit of detection of 615 IU/ml. The epidemiology and clinical outcomes of hepatitis B and C co-infection in EuroSIDA have been published previously [20]–[22].

For the present study all patients positive for anti-HCV antibodies and/or HBsAg with at least one available plasma sample were included.

#### Study end points

The primary end point was the composite of liver-related death or the development of hepatic encephalopathy. To assess the specificity of the predictive potential of HA for liver related clinical outcomes, non-hepatic deaths as well as new, non-recurrent AIDS events were included as secondary end points. Causes of death were determined using the Coding of Death in HIV (CoDe) algorithm [23]. Hepatic encephalopathy was defined as grade 3 or 4 based on the West Haven Criteria of Altered Mental Status In Hepatic Encephalopathy [24]. The diagnostic criteria are specified in the EuroSIDA list of definitions of clinical events.

#### Hyaluronic acid

All quantitative HA measurements were done centrally using a commercial enzyme linked binding protein assay (Corgenix, Colorado, USA) with a HA range in a healthy population between 0–75 ng/mL. The dynamic range of the assay is from 10 ng/mL to 800 ng/mL (package insert, Corgenix). Samples with HA concentrations greater than 800 ng/mL were diluted and re-assayed. The first available centrally stored plasma sample, after the patient was tested positive for HCV or HBV, was identified and HA was measured in duplicate according to the manufacturer’s specifications.

All patients with available samples that developed a liver-related event during follow-up had HA measured in the last available sample prior to their event. A control group was selected by assigning each patient, who did not develop a liver-related event, a random number in Excel. The list was then sorted according to the number and every sixth patient was selected as a control. The technicians, who performed the HA measurements were blinded to the study outcomes.

### Statistical Methods

Patients were divided into two groups. The first group consisted of patients with either chronic hepatitis B (HBsAg positive; denoted HBV+) and/or chronic hepatitis C (anti-HCV/HCV-RNA positive; denoted HCV+) at baseline. The second group consisted of patients who were HBsAg negative and had serological evidence of cleared HCV infection (anti-HCV positive/HCV-RNA negative). Characteristics of patients were compared between groups using the chi-squared test for proportions or the non-parametric, Wilcoxon or Kruskall-Wallis test for continuous variables. The incidence of liver related events was calculated by dividing the number of events by the patient-years of follow-up (PYFU). Patients were followed from baseline, defined as the date of the HA measurement, to the clinical event or to last follow-up for those patients who did not experience a clinical event. Only one event per patient was included in analyses. Kaplan-Meier figures were used to estimate the proportion of patients with a liver-related event in different HA strata and Poisson regression was used to investigate the relationship between baseline levels of HA and progression to a liver related event. Potential explanatory factors, in addition to baseline HA, included age, gender, risk group, ethnic origin, date of recruitment to EuroSIDA, date of baseline, exposure to antiretrovirals, CD4 count, HIV-viral load, region of Europe, and prior AIDS diagnosis. Any factor that was significant in univariate analyses (p<0.1) was included in multivariate analyses.

The change in HA for patients with a liver-related event and the control group was calculated and standardised for the follow-up time between the measurements to express the change in HA per year of follow-up. This measurement was quite variable and therefore logistic regression was used to describe which factors were associated with a single standard deviation increase in HA using the standard deviation of change from all cases and controls. Similar factors as described above were included as potential confounding variables.

All analyses were performed using SAS (Statistical Analysis Software, Cary, NC, USA, Version 9.3).

## Results

### Baseline Characteristics

Overall 1252 patients were included, and their characteristics at time of the first HA measurement (i.e. baseline) is shown in table 1. In total, 1090 (87.1%) were HCV+ (n = 758), HBV+ (n = 256) or both HBV+ and HCV+ (n = 76), while 162 (12.9%) were anti-HCV antibody positive/HCV-RNA negative. The median HA level for all patients was 33.9 ng/mL (interquartile range [IQR] 17.9–69.1), and was higher in patients with chronic HCV infection (37.7 ng/mL (18.8–76.8)), than in patients with chronic HBV infection (31.4 ng/mL (18.0–63.4)) or cleared HCV infection (27.5 ng/mL (15.7–51.1)).

**Table 1:** Baseline characteristics of all patients and stratified according to hepatitis status.

		All		HBsAg Pos and/or HCV Ab Pos and HCV RNA pos		HBsAg Neg and HCV Ab pos and HCV RNA neg		p
		N	%	N	%	N	%	
All		1252	100	1090	87.1	162	12.9	
Gender	Male	894	71.4	788	72.3	106	65.4	0.070
	Female	358	28.6	302	27.7	56	34.6	
Race	White	1101	87.9	954	87.5	147	90.7	0.24
	Other	151	12.1	136	12.5	15	9.3	
Risk	MSM	232	18.5	206	18.9	26	16.1	0.24
	IDU	756	60.4	648	59.5	108	66.7	
	Het	153	12.2	134	12.3	19	11.7	
	Other	111	8.9	102	9.4	9	5.6	
Region	S	290	23.2	252	23.1	38	23.5	0.95
	C	364	29.1	317	29.1	47	29.0	
	N	276	22.0	238	21.8	38	23.5	
	E	322	25.7	283	26.0	39	24.1	
AIDS	Yes	361	28.8	303	27.8	58	35.8	0.036
ARVs	None	76	6.1	67	6.2	9	5.6	0.94
Ever	ART	121	9.7	106	9.7	15	9.3	
Started	cART	1055	84.3	917	84.1	138	85.2	
		Median	IQR	Median	IQR	Median	IQR	
Age	Years	38	33–43	38	33–43	38	33–44	0.57
CD4	/µL	452	257–648	453	260–647	442	230–649	0.85
CD4 nadir	/µL	136	51–240	139	53–240	105	34–225	0.064
HIV-RNA	Log_10_ copies/ml	1.6	1.6–2.3	1.6	1.5–2.3	1.6	1.6–2.6	0.43
HA date	Month/year	8/00	7/98–12/01	8/00	7/98–12/01	3/00	5/98–10/03	0.84
HA	ng/mL	33.9	17.9–69.1	35.5	18.2–70.5	27.5	15.7–51.1	0.0054

HBsAg: Hepatitis B surface antigen; HCV Ab: anti-HCV antibody; MSM: men who have sex with men; IDU: intravenous drug use; Het: heterosexual; S/A: south/Argentina; C: central; N: north; E: east; ARVs: antiretroviral drugs; ART: antiretroviral therapy; cART: combination antiretroviral therapy; HA: hyaluronic acid; IQR: interquartile range.

### Baseline HA and Risk of a Clinical Progression

During a median follow-up of 8.2 years (IQR 4.7–11.5 years) 84 (6.7%; 52 liver-related death and 32 hepatic encephalopathy) patients developed a liver-related event (LRE; hepatic encephalopathy or liver-related death). Eight and seven LREs occurred among patients who were HBsAg positive/anti-HCV negative and anti-HCV positive/HCV-RNA negative/HBsAg negative, respectively. Among the eight HBV patients, five were on tenofovir based ART at the date of death. 138 (11.0%) patients died from non-liver-related causes while 138 (11.0%) developed AIDS during follow-up. In unadjusted analysis, the incidence rate for all three end-points was similar when comparing patients with chronic viral hepatitis with the “HCV antibody positive/HCV-RNA negative” group (p>0.3 for all comparisons).

**Figure 1:**
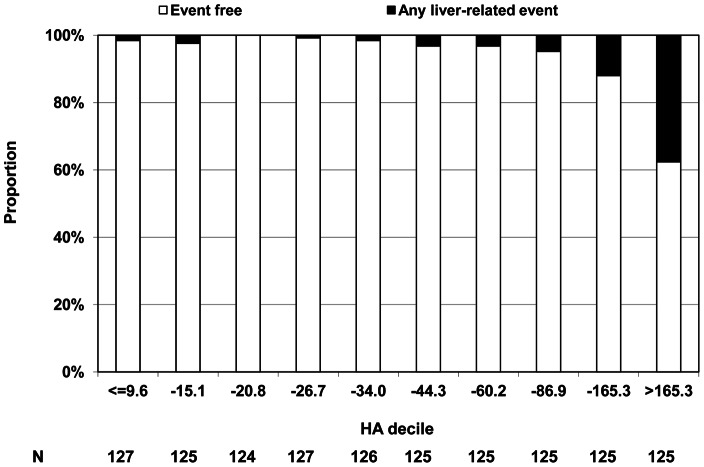
Distribution of plasma hyaluronic acid levels and any-liver related events during follow-up.

For patients who developed an LRE during follow-up the median HA at baseline was 221.6 ng/mL (IQR 74.9–611.3), while it was 31.8 ng/mL (IQR 17.2–62.6) for patients who did not experience an LRE. For patients with chronic viral hepatitis who developed an LRE (n = 72) the median HA was 229.1 ng/mL (IQR 78.5–537.3), whereas it was 124.5 ng/mL (74.9–1025.4) for the seven patients with cleared HCV infection who developed an LRE (p = 0.72). One of the seven patients had received interferon-based treatment prior to inclusion in the study. Figure 1 shows the distribution of baseline HA levels for all patients divided in to deciles; the proportion of patients experiencing any LRE was low with low HA. 47 (37.7%) events occurred with a baseline HA >165.3 ng/mL, while only 6 out of 503 (1.2%) with a baseline HA <26.7 ng/mL experienced an event.

**Figure 2:**
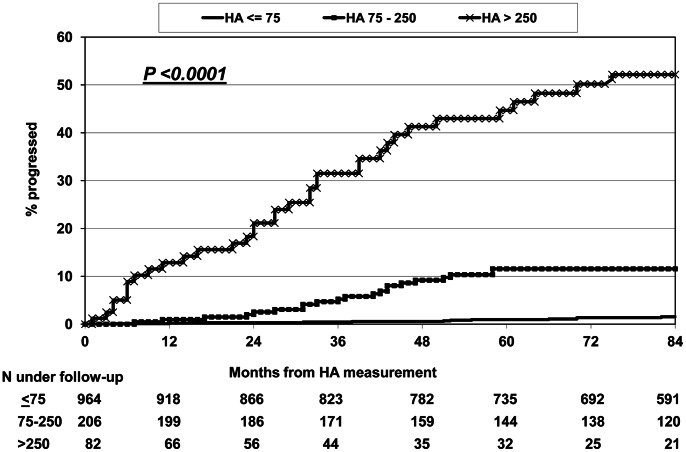
Kaplan Meier progression to any liver-related event according to baseline plasma hyaluronic acid level (ng/mL).

Patients were then divided into three groups depending on whether the HA level was below the upper limit of normal ≤75 ng/mL), moderately elevated (75–250) or markedly elevated (>250), and the risk of a LRE was estimated (Figure 2). Those with normal levels of HA had a cumulative 5-year risk of experiencing an LRE of 1.0% (95% CI 0.3–1.6%), while the 5 year risk for moderately elevated HA was 11.6% (6.9–16.2) and for markedly elevated HA 44.7% (95% CI 32.7–56.7%, p<0.0001). The risk of contracting a LRE increased gradually and most events occurred more than 6–12 months after the time when the HA level was determined (Figure 2).

**Figure 3:**
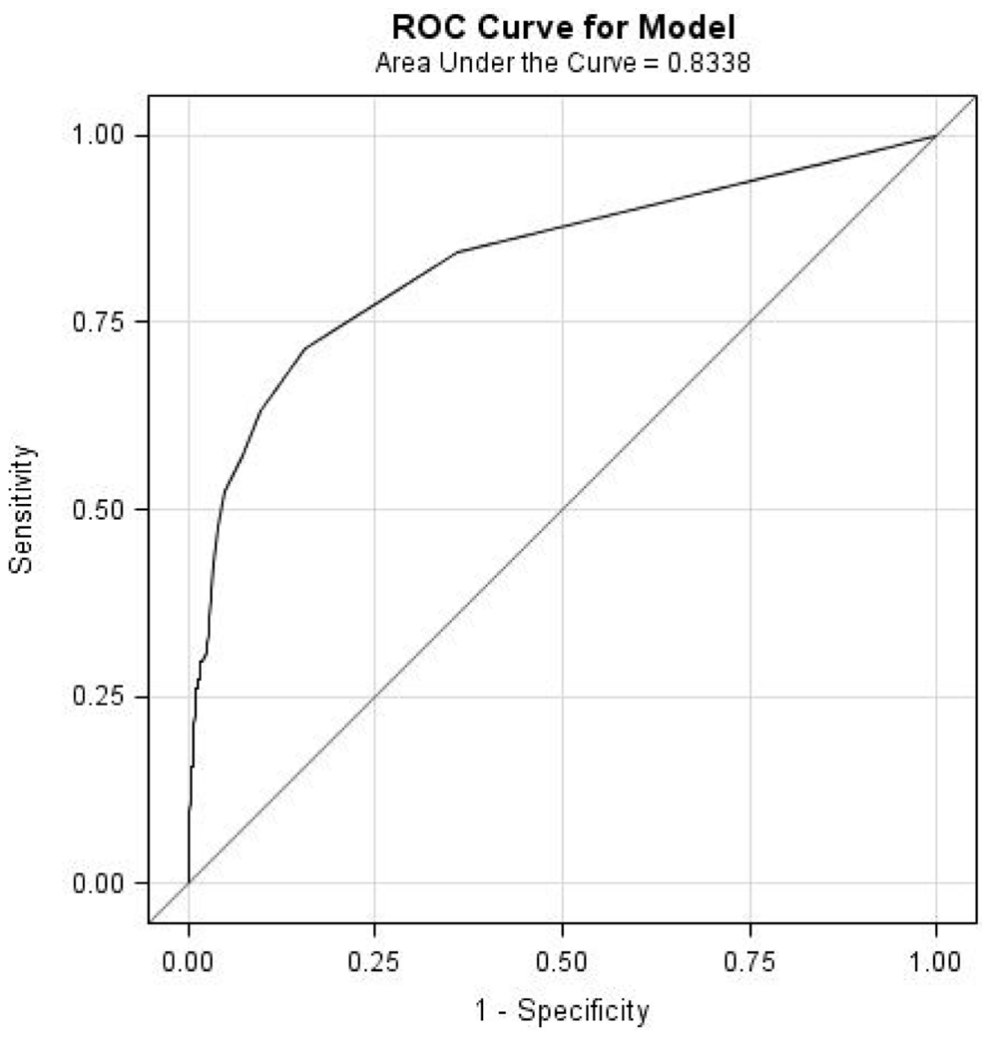
Receiver operating characteristic curve for the ability of plasma hyaluronic acid to predict liver-related events.

The prognostic performance of HA to predict LRE, as estimated by the area under the ROC curve, was 0.83 (95% CI 0.79–0.88) (Figure 3). A HA cut-off value of 100 ng/mL provided the highest sum of sensitivity (69.03) and specificity (87.0), while the positive predictive value (PPV) and negative predictive value (NPV) were 27.6 and 97.5, respectively. Changing the cut-off value to 250 ng/mL increased the PPV to 46.3, but only decreased the NPV to 96.1. There were no differences in these performance estimates when comparing patients with chronic viral hepatitis with those with cleared HCV infection (results not shown).

**Figure 4:**
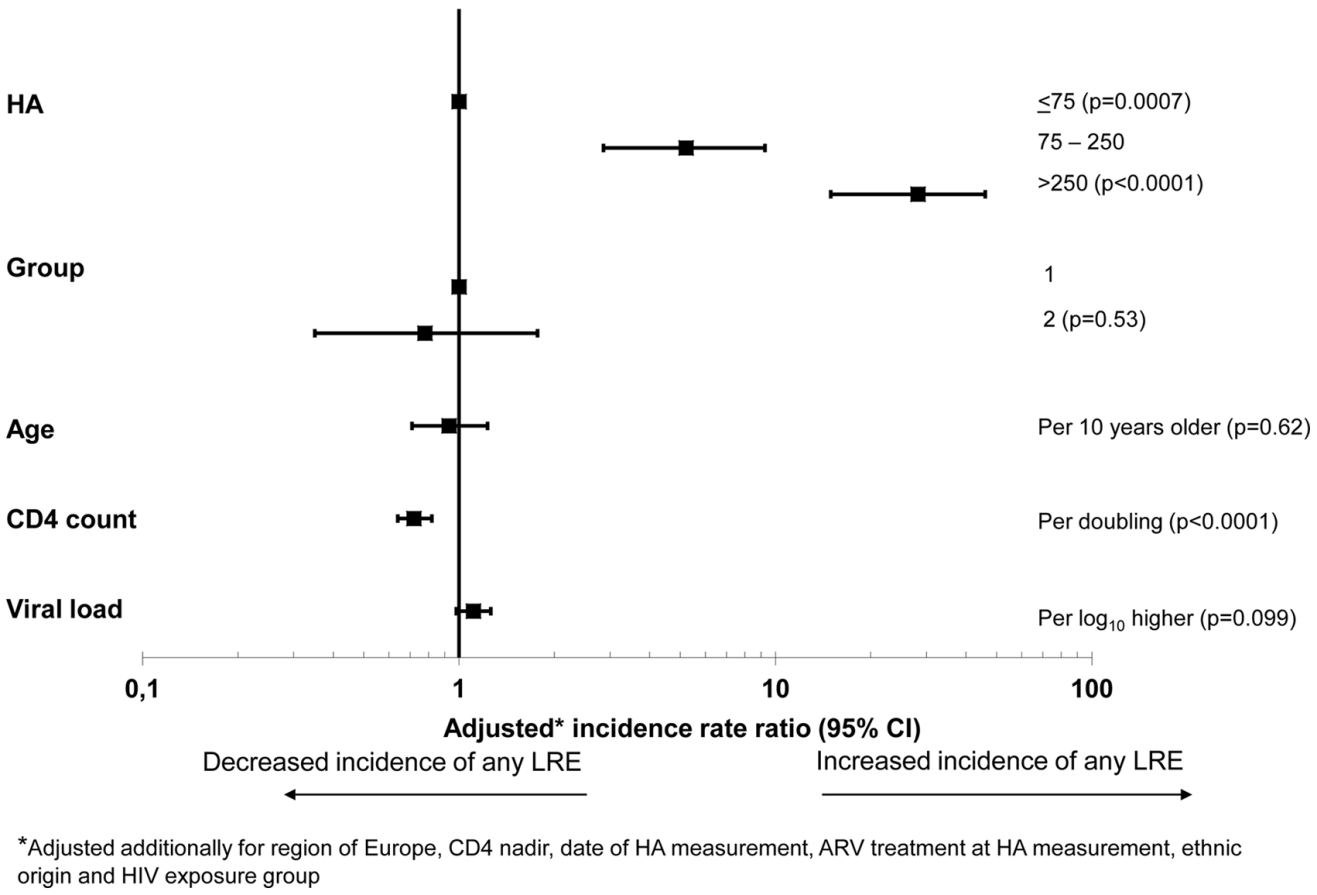
Adjusted incidence rate ratios of any liver-related events. Note: The horizontal lines indicate 95% confidence intervals; HA: hyaluronic acid; HBsAg: hepatitis B surface antigen; HCVab: anti-HCV antibody; Group 1: HBsAg positive and/or anti-HCV/HCV-RNA positive; Group 2: anti-HCV positive/HCV-RNA negative.

**Figure 5:**
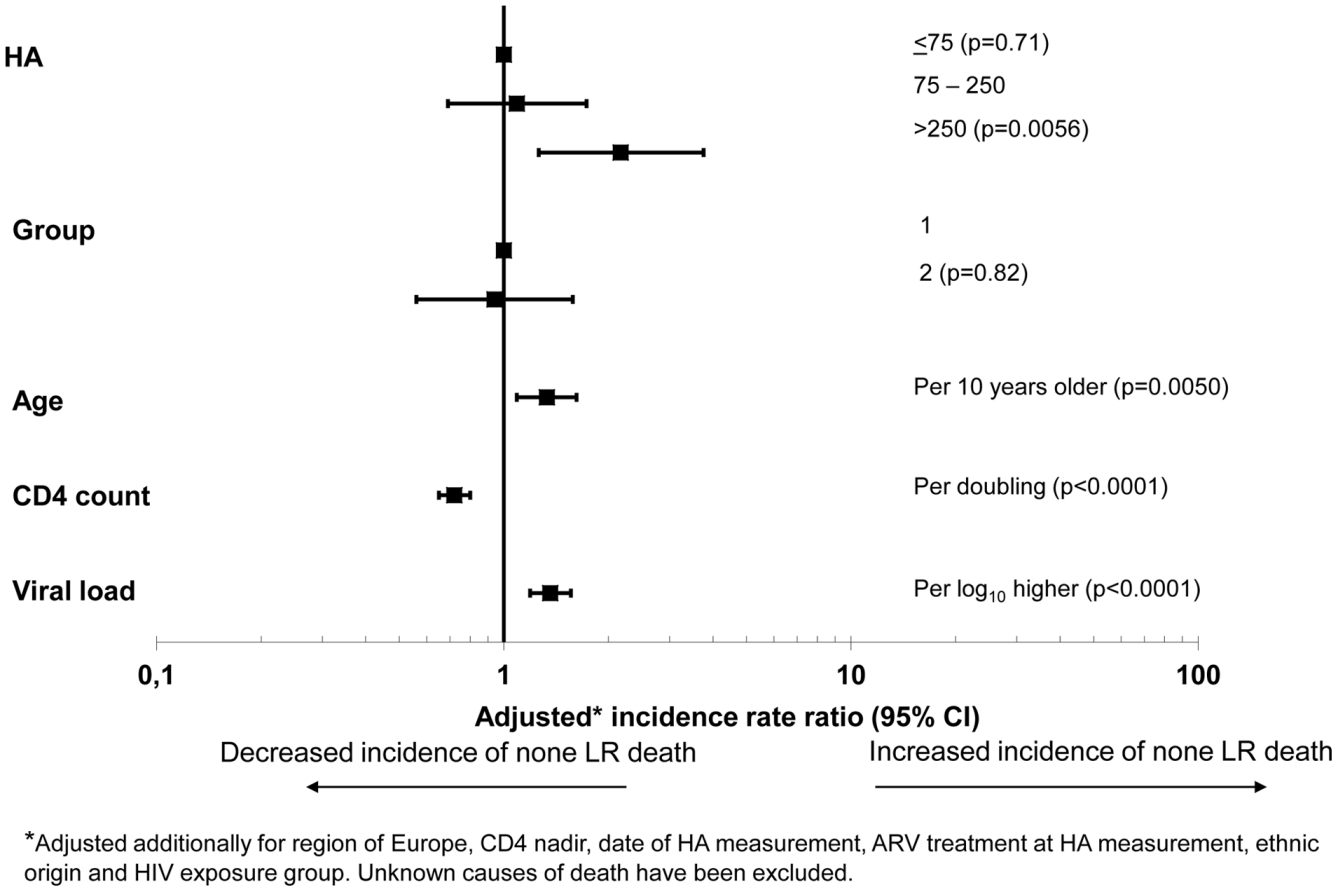
Adjusted incidence rate ratios of any non-liver-related events. Note: The horizontal lines indicate 95% confidence intervals; HA: hyaluronic acid; HBsAg: hepatitis B surface antigen; HCVab: anti-HCV antibody; Group 1: HBsAg positive and/or anti-HCV/HCV-RNA positive; Group 2: anti-HCV positive/HCV-RNA negative.

The multivariate incidence rates ratios (IRR) for any LRE and non-liver-related death are shown in figure 4 and 5, respectively. Age, baseline CD4+ count, HIV viral load and HA level (categorised as ≤75, 75–250 and >250 ng/mL) were significant predictors (p<0.1) of any LRE in the univariate analysis, but HA was by far the strongest predictor. After adjustment only baseline HA level, and CD4+ count were significant predictors of LRE, and HIV-viral load was of marginal significance. Compared to patients with normal HA levels, those with moderately elevated HA levels had a 5-fold increase of any LRE (IRR 5.22; 95% CI 2.86–9.26, p = 0.0007), whilst those with markedly elevated HA level had almost a 30-fold increased incidence of any LRE (IRR 28.22; 95% CI 14.95–46.00, p<0.0001). Sensitivity analyses using time-updated variables (CD4+ cell count, HIV viral load and time of cART initiation), adjusting for HCV genotype or censoring patients at starting any interferon-based therapy (n = 182) did not change the IRRs significantly (results not shown).

In a sensitivity analysis, excluding patients with HBV infection, the multivariate IRRs for LRE were similar. With patients with normal HA levels as reference, the IRR (95% CI) was 8.37 (4.09–17.12; p<0.0001) and 30.63 (15.14–59.46; p<0.0001) for patients with HA elevated moderately or markedly, respectively. Only eight and seven LRE occurred among HBV+ and patients with resolved HCV infection, respectively, which precluded similar analyses for the two groups.

For non-liver-related death both age, CD4, HIV viral load and markedly (but not moderately) elevated HA levels were significant predictors of this outcome, although the risk for those with markedly elevated HA levels was modest (IRR 2.17; 95% CI 1.26–3.76, p = 0.0036) (Figure 5). Conversely, HA levels failed to predict the occurrence of non-fatal or fatal AIDS events all together (results not shown).

### HA during Follow-up for LRE and Controls

43 (51.2%) patients that developed an LRE during follow-up had HA measured in their last available sample prior to their event (cases). 172 (14.7%) other patients without a LRE were included as random controls. Characteristics of cases included and excluded from this analysis were comparable, except that included cases had the first HA measured earlier than the excluded cases (median dates September 1998 versus March 2000, p = 0.034). Similarly included controls had an earlier date of HA measurement compared to excluded controls (median dates September 1999 versus February 2001, p<0.0001). Apart from the difference in HA when comparing the included cases and included controls (medians 230.5 ng/mL versus 30.5, p<0.0001), cases were older (median age 41 versus 37 years, p = 0.0012), had higher HIV RNA viral load at measurement of HA (medians 2.7 log_10_ copies/ml versus 1.6 log_10_ copies/ml, p<0.0001) and had HA measured earlier (median date September 1998 versus September 1999, p = 0.009). The median time between first and last HA measurement was 2.3 years (IQR 1.3–4.0 years) and did not differ when comparing cases and controls (p = 0.063).

Median change in HA/year was 111.1 ng/mL (IQR 0.8 to 287.5) for the cases compared with 1.0 ng/mL (IQR –5.1 to 8.2) for the controls (p<0.0001). 12 (28.6%) of the cases had an increase in HA >210 ng/mL (1 SD increase) compared to 6 of the controls (3.6%, p<0.0001).

For the patients who developed a LRE the median time from last HA measurement to the event was 8 months (IQR 4–16). Those who progressed to an event more quickly after last HA (i.e. ≤8 months, 21 patients) had a similar baseline HA (median 270.3 ng/mL, IQR 128.6–701.2) compared to those who progressed to a liver related event more slowly (ie >8 months, n = 21, median 227.6 ng/mL, IQR 129.4–380.4, p = 0.55). However, in those who progressed more quickly, the HA immediately before the event was higher (median 922.2 ng/mL, IQR 605.1–1236.5 vs. 402.2 ng/mL, IQR 135.7–627.3, p = 0.0066), and there was a greater change in HA from first to last measurement (median change per year 181.3 ng/mL, IQR 104.8–505.3 vs. 7.2 ng/mL, IQR –19.8–115.9, p = 0.0063).

Logistic regression was used to look at odds of >1 SD (210 ng/mL) increase in HA. In the univariate analysis cases had almost an 11-fold increased odds of >1 SD in HA (odds ratio [OR] 10.87, 95% confidence interval [CI] 3.79–31.19, p<0.0001). In multivariate analysis, predictors of >1 SD change in HA were lower CD4 counts at HA measurement (per doubling) (OR 0.67, 95% CI 0.51–0.88, p = 0.0042) and being a case (OR 6.77, 95% CI 2.38–19.32, p = 0.0003).

## Discussion

Many non-invasive methods for diagnosing liver fibrosis have been evaluated in recent years, primarily in patients with chronic HCV infection. With few exceptions, these studies have been cross-sectional, focusing on staging hepatic fibrosis and lacking clinical end-points. The Model for End-Stage Liver Disease (MELD) and the Child-Pugh score are normally used to assess the prognosis of patients with end-stage liver disease [25], [26], but have not been validated in patients with early stages of liver disease. To date, the long term prognostic value of fibrosis markers have been evaluated in only three studies of HCV mono-infected patients [15], [16], [27], [28] and in a cohort of both HCV mono-infected and HIV/HCV co-infected patients [29]. Our study is the first to evaluate the prognostic value of a biomarker of fibrosis in a large cohort of viral hepatitis co-infected HIV patients.

The median plasma HA level for all patients was 33.9 ng/mL, which is comparable to findings from studies of other co-infected populations [30], [31], but lower than in some studies that only included patients who had a liver biopsy performed [17], [32], which could have selected for patients with more advanced liver fibrosis. Plasma HA level was a very strong predictor of later development of hepatic encephalopathy or liver-related death. HA level in the first available plasma sample after the viral hepatitis diagnosis was considerably higher in patients who later experienced an LRE compared to patients that remained free of such an event. The 5-year risk of developing an LRE was around 45% for patients with a baseline HA >250 ng/mL, whereas it was 12% in patients with HA between 75 and 250 ng/mL and only 1% if HA was ≤75 ng/mL initially. The optimal cut-off level, as estimated by ROC curve analysis, was 100 ng/ml, which gave a PPV and NPV of 27.6 and 97.5 for an LRE, respectively. Analyzing patients with HIV/HCV co-infection separately gave comparable results. Seven patients with cleared HCV infection (one due to HCV treatment) and no other known risk factors for liver disease developed an LRE. Their baseline HA level was lower, but not statistically different, than in patients with chronic hepatitis who experienced an LRE. This underscores the importance of also evaluating the extent of liver fibrosis in this patient group, and closely monitor and explore other risk factors for liver disease in those with signs of significant fibrosis.

To evaluate the predictive ability of changes in plasma HA over time we tested the last available plasma sample in patients who later developed an LRE and compared them with a control group of random patients who did not develop a LRE. Although not all patients who developed an LRE or controls had stored samples, we were able to assess changes in HA in a substantial proportion of the cases. Patients with an event had an annual increase in median HA level of around 111 ng/mL compared to 1 ng/mL for the controls. Of note however, almost one quarter of patients who developed an event had decreasing HA during follow-up. Serial measurements will likely reduce the variability and further improve the prognostic information derived from measuring HA. Interestingly, we also found that higher CD4 counts were associated with a reduced risk of substantial increases in HA levels during follow-up, consistent with the assumed protective role of high CD4 counts on the risk of progression of liver fibrosis in co-infected patients [33].

The HA assay is very robust with an average intra- and inter-assay coefficient of variation around 4% and 6%, respectively (package insert, Corgenix). Like all other non-invasive fibrosis markers, HA is not liver specific. Increased HA levels can also be seen during synovial inflammation and cartilage destruction in rheumatoid arthritis and other connective tissue diseases, due to increased production and passage of HA into the circulation [34]. HA may increase after intake of food, most likely due to an increase in post-prandial intestinal and thoracic duct lymph-flow with increased input of HA in to the circulation [35], [36]. The patients in EuroSIDA are not required to be fasting before blood samples are drawn. Thus, it is possible that the low positive predictive value we observed, but also decreases in HA during follow-up in around a quarter of all patients who developed an LRE, to some extent could be due to the influence of food intake.

HA is not measured routinely in most biochemistry laboratories, and before introducing HA as a routine test in clinical practice, further studies with clinical end-points validating HA against liver-biopsy and other non-invasive methods, including biomarkers that use routine laboratory tests, are required. Future studies should also define the optimal cut off values to maximise the predictive value of HA in routine clinical settings and also investigate factors influencing the longitudinal dynamics of HA. However, the present study indicates that HA could be a useful biomarker to estimate the long-term risk of liver disease. Patients with low (<75 ng/mL) and stable plasma HA levels would have very little risk of clinical progression over the next five years. Patients with elevated or increasing plasma HA levels should be followed clinically more closely and their risk of liver disease should be assessed with more specific tests.

Our findings also have implications for future research efforts focused on assessing the impact of impairment of liver function on various types of clinical outcomes in HIV positive populations. For example, impairment of liver function may contribute to dysregulation of coagulation in HIV positive persons [37] which is associated with mortality [38]. To pursue these research questions, it has proven to be a challenge to reliably evaluate the degree of liver impairment in large cohorts of patients followed in multiple sites, as liver biopsy is infrequently performed, and Fibroscan is only available in selected sites. Additionally, the interpretation of other non-invasive measures of fibrosis such as APRI and FIB-4 is complicated by the fact that some of the parameters used in these scores are affected by HIV-specific factors [39], [40]. Comparative studies in co-infected patients have shown HA to be superior to APRI and FIB-4 in diagnosing advanced fibrosis [14], [17], [18]. Hence, soluble biomarkers that assess impaired liver function via other mechanisms are attractive research tools. Our findings support that HA is a suitable candidate for this purpose. Indeed, our data was part of the reason why the INSIGHT (International Network for Strategic Initiatives in Global HIV Trials) network chose HA as the most suitable marker of liver fibrosis [31], [41].

This study has some limitations. Until 2010, hepatic encephalopathy and liver-related deaths was the only manifestations of decompensated liver cirrhosis that is routinely collected in EuroSIDA, and it is likely therefore that the study underestimates the burden of other types of non-fatal end-stage liver disease events. It is possible that the positive predictive value of HA would have increased had ascertainment of such events also been available. Only 43 out 84 patients with an LRE had a follow-up sample available. It is likely that those who died most quickly had no follow-up sample, thus leading us to underestimate the rate of change in HA. Also, we were not able to compare HA with MELD or other biomarkers of fibrosis like APRI and FIB-4, since INR and platelet levels have only been collected routinely in EuroSIDA since 2006. Likewise, we did not have access to information on alcohol consumption or liver biopsy results from the fraction of patients who had this procedure performed.

In summary, this large cohort study demonstrates that baseline HA was a strong predictor of later development hepatic encephalopathy or liver-related death in HIV-1 patients co-infected with HBV and/or HCV. Patients, who during follow-up experienced a liver-related event, had higher annual increase in HA compared to patients without an event, while a high CD4 cell count was associated with a less rapid increase in HA. Therefore, plasma HA may be useful, either alone or in combination with other non-invasive methods, to monitor progression of liver disease and risk of complications in patients with chronic viral hepatitis.
